# The Human Body as a Machine

**Published:** 1889-03

**Authors:** Walton Harrison


### THE HUMAN BODY AS A MACHINE.

BY WALTON HARRISON.

It has often been stated, figuratively, that the human body is a machine. Probably it has never been claimed that the human body, or that of an animal, or a plant, is a machine based exclusively upon scientific laws—a machine in a mechanical sense ; yet there is much evidence corroborative of the fact that such is undoubtedly the case.

All matter is divided into three great classes, called respectively the animal, mineral and vegetable kingdoms ; this is an arbitrary classification, however, as the individuals comprising the animal and vegetable kingdoms are compounds of substances belonging to the mineral kingdom. For instance, a living oak tree is a vegetable ; yet it is composed entirely of oxygen, hydrogen, carbon and other minerals. The human body is likewise composed entirely of elementary mineral substances, such as iron, oxygen, calcium, hydrogen and carbon. These elements are of the same kind as those which, singly or combined, compose each and every terrestrial object ; they are inanimate, insensible and inorganic ; they are the simplest of all substances. The human body, therefore, consists of such substances as those which compose ordinary machinery of any kind—the only difference being in their selection, proportion and classification. Surely nobody with intelligence will state that the oxygen and hydrogen which are the principal ingredients of animal flesh are at all different from the oxygen and hydrogen which are found in the ocean, or which are necessary in the boiler of a locomotive. The iron axle of a clock wheel may be reduced to palatable form, and introduced into the system of a man ; it will not there remain as a foreign substance, but will be taken into the blood and became a part of the living man. So also may a chemist take into his laboratory, a few gills of healthy human blood, and separate therefrom a bit of metallic, malleable, commercial iron, capable of rendering service as a working part of the clock. The body of a full grown man, animal or vegetable is not the same body which was inherited at the commencement of life ; changing particle by particle, in accordance with the wonderful law of evolution, it has become another physical being. Its substance, therefore, is interchangeable.

If then, the human body is composed exclusively of simple elements, similar, in all respects to the elements which compose a locomotive (a fact admitted by every scientist in the world), whence does it derive its animation, its power of acting, of performing work ? Whence is the origin of thought ? Why do the various organs perform their functions ?

How can the body repair its injuries, change its substance, and reproduce other bodies? Obviously, these phenomena can only be brought about in one of two ways; (1) either a series of miracles are constantly being performed, or (2), through the direct agency of natural laws, similar to and including those already known to man. The analogy of all nature would indicate, that it is through the direct agency of natural laws, and through that source alone, that each and every function of the body is performed. Perhaps some of the laws governing the body will never be understood, but they exist, nevertheless. As the substance of the body is identical with the substance of all nature, so the laws, properties, and conditions governing the body so far as known, are identical with the laws, properties and conditions of all other matter; for instance, the body is influenced by the law of gravitation, is held together by the attraction of cohesion, and when in motion acquires momentum.

The various parts of the body, severally and collectively, move and perform work because of the same law which causes the various portions of the locomotive to perform work—the first law of mechanics, which is as follows: "A body at rest will forever remain at rest *unless acted upon by a motive power*." The body, then, is only the servant of a motive power, just as the engine is the servant of heat. The brain, being a part of the body, is a combination of inanimate substances; it cannot, therefore, move or perform any manner of work unless acted upon by a motive power—else we have the first instance of a violated mechanical law. How a motive power acting upon the brain can cause the latter to produce thought will never, perhaps, be thoroughly understood—neither is it understood why a motive power acting upon the band-wheel of a dynamo causes that machine to put forth electricity. The human body, like any other machine, puts forth energy when acted upon by its motive power, consumes (or rather changes) materials in so doing, and stops running when sufficiently disabled.

There is not, in the body, a movement which is inconsistent with mathematics; there is not a bone which is incompetent to stand the test of architecture; there is not a tube, or liquid, or valve, or pumping fixture inconsistent with the laws of hydraulics; every limb is made up of mechanical appliances; the ear will stand the test of acoustics, and the eye, as its name indicates, is an optical instrument. The body will stand every test to which, as a compound machine, it could be subjected. Like the more imperfect machines constructed by human genius it works, in part and as a whole, in accordance with natural laws framed centuries ago by the Creator, it is therefore a machine, and a machine only.

